# Establishment and application of a visual nucleic acid detection method for parvovirus

**DOI:** 10.1186/s12917-025-04993-5

**Published:** 2025-10-02

**Authors:** Shaoting Weng, Mingliang Zhang, Yilin Zhu, Qiaoying Song, Yifan Zhang, Sumei Zhang, Kunpeng Zhang, Sen Lin, Xinying Ji, Yao Wang

**Affiliations:** 1 Department of Biotechnology, Anyang Institute of Technology, Anyang, Henan 455000 China; 2 College of Veterinary Medicine, Henan Agricultural University, Henan, 450000 China; 3 College of Basic Medical Sciences, Henan University, Henan, 475000 China; 4 Anyang Kindstar Global Medical Laboratory Ltd, Anyang, Henan 455000 China

**Keywords:** Parvovirus, VP2 gene, T7 endonuclease I, Fluorescent probes, Recombinase polymerase amplification, Visualization

## Abstract

Parvoviruses pose a significant threat to the livestock breeding industry, leading to high morbidity and mortality rates. Rapid, accurate, and convenient detection methods are crucial for early intervention and control of parvovirus infection. This study established a novel visualized parvovirus detection method based on nucleic acid-mismatched enzyme detection—NMED. This method targets a conserved region of the Canine Parvo Virus** (**CPV**)** VP2 gene by utilizing specific fluorescent reporter probes and Recombinant Polymerase Amplification༈RPA༉ primers. Upon recognition of the target sequence by the reporter probe, T7 endonuclease Ⅰ (T7E Ⅰ) is activated, cleaving the probe and enabling detection under UV light or via the use of a fluorescence reader. Our research results show that we have successfully established a novel nucleic acid-based method for detecting CPV. This method can detect nucleic acid within 50 min. It exhibits high specificity, demonstrating no cross-reactivity with common canine pathogens. The method has a lower limit of detection of 1–10 copies/µL. The three replicates consistently yielded identical results, indicating high stability. Compared with qPCR, the NMED method achieved a concordance rate of 90.63%—93.75% for 32 suspected CPV samples. In conclusion, this study developed a convenient, efficient, visual nucleic acid detection method for CPV. The establishment of NMED has provided a new method for detecting parvovirus, which is highly important for the timely prevention and control of parvovirus infection and the reduction of livestock mortality.

**Supplementary Information:**

The online version contains supplementary material available at 10.1186/s12917-025-04993-5.

## Background

Parvoviruses, as a group of widely distributed and pathogenic tiny virus, have caused highly lethal animal infectious diseases (such as canine hemorrhagic enteritis and swine reproductive disorder syndrome), which have posed continuous biosecurity challenges to the livestock economy and the prevention and control system of zoonotic diseases [1–3]. According to the latest classification by the International Committee on Taxonomy of Viruses (ICTV), parvoviruses are divided into 11 genera, with the genus Parvovirus containing members responsible for highly contagious and fatal diseases in various animal species. Notable examples include canine parvovirus (CPV), feline panleukopenia virus (FPV), and porcine parvovirus (PPV) [4]. These viruses exhibit short incubation periods and rapid transmission, leading to severe gastroenteritis or reproductive disorders and posing a serious threat to livestock [5, 6]. The widespread presence of these viruses and their potential for cross-species transmission highlight the need for robust preventive measures and vigilant surveillance to mitigate the impact of parvovirus infections globally.

Parvoviruses are a class of small viruses with a linear single-stranded DNA genome, lacking an envelope, and possessing a capsid. The viral particles are icosahedral in symmetry and approximately 25 nm in diameter, with a genome size of approximately 5–6 kb [7]. The genome possesses palindromic hairpin structures at both ends, which are associated with viral replication [8]. The parvovirus genome harbors two open reading frames (ORFs) that encode a nonstructural protein (NS) and a capsid protein (VP). Notably, the capsid protein VPis highly antigenic, contributing to the strong resistance and transmission capability of the virus [9]. Currently, there is no effective drug that can completely cure parvovirus infection, and early treatment is crucial for controlling the spread of the virus and improving cure rates [10]. Therefore, rapid and accurate diagnosis of parvovirus is highly important for the prevention and treatment of this disease.

Traditional parvovirus detection methods include mainly qPCR, virus antigen detection, virus isolation and culture, and immunochromatographic assays [11–14]. However, these methods suffer from drawbacks such as long detection times, complex operations, poor specificity, low sensitivity, high detection cost, and the need for specific instruments and equipment, which significantly limits their practical application. Therefore, the development of a rapid and visual parvovirus detection method is highly important. In recent years, many research efforts have focused on developing point-of-care testing (POCT) tools for the diagnosis of infectious diseases that can be used under field conditions. POCT methods or devices would allow for the performance of pathogen diagnostics on rural farms or in resource-limited settings [15]. Many detection methods based on isothermal nucleic acid amplification technologies, including loop-mediated isothermal amplification (LAMP), recombinase polymerase amplification (RPA), and nucleic acid sequence-based amplification (NASBA), have been developed for the detection of animal viruses [16]. Among them, RPA technology has been developing rapidly and widely adopted. Examples include the RPA-nfo assay [17], the RPA-fpg assay [18], and the RPA-exo assay [19]. Unlike traditional PCR techniques, these methods do not require complex instruments and eliminate the tedious steps of temperature cycling. They enable rapid DNA or RNA amplification at low temperatures (typically 39 °C), producing amplified products within 30 min. The results can be visualized by observing the changes in fluorescent signals released from the reaction products, offering advantages such as speed, sensitivity, and visual results.However, these methods are highly influenced by reaction conditions, have low specificity, and are prone to false positives [20]. Currently, the prevailing viral detection method internationally is CRISPR molecular diagnostic technology (CRISPR-based diagnostic, CRISPR-Dx). It is a type of nucleic acid detection method based on the nonspecific collateral cleavage of Cas12/13 combined with isothermal amplification techniques, lateral flow assays (LFAs) or electrochemical biosensors.These systems are based on Cas12/13 proteins, combining Cas12/13 with RPA, using collateral cleavage activity to release the fluorescence signal of nonspecific fluorescent probes, realizing molecular diagnosis [21]. These methods offer advantages such as signal amplification, convenience, high efficiency, and the absence of expensive equipment. However, it also has certain limitations, such as the high requirement for crRNA design and synthesis, the nonspecificity of Cas12/13 enzyme-mediated cleavage, and the interference of off-target effects in sequence detection [22].

**Fig. 1:**
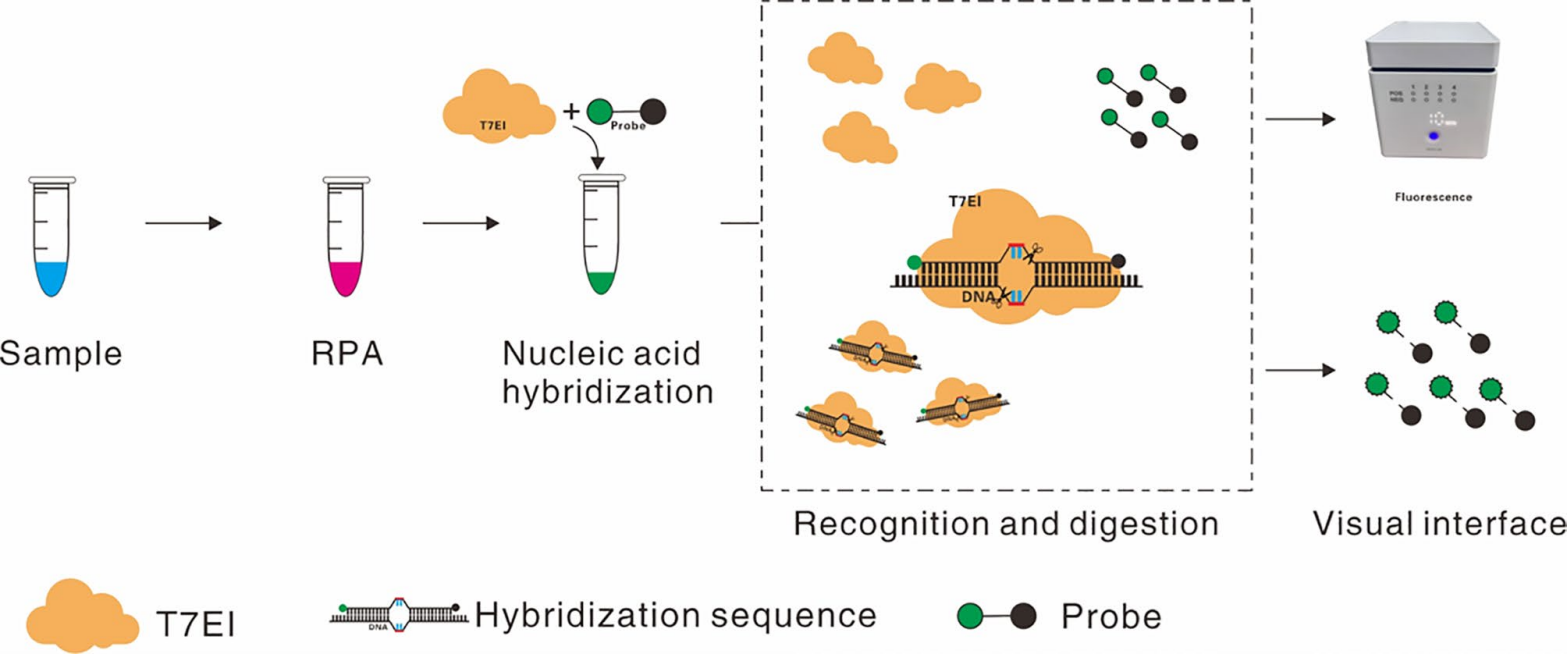
Workflow of the NMED assay. The whole detection process: After nucleic acid lysis, the target sample undergoes RPA amplification, generating a large amount of DNA containing the target sequence. Specific 6-FAM-HBQ1 fluorescently labeled probes (containing two mutated bases) are then used to recognize the target sequence through nucleic acid hybridization. If the probe binds to the target sequence, it activates the endonuclease activity of T7E Ⅰ at the mismatched site, cleaving the fluorescent probe and releasing the FAM fluorophore. Finally, visual determination can be performed with a simple device that excites fluorescence. In contrast, if the probe fails to recognize the target sequence, T7E Ⅰ cannot cleave, and the FAM fluorophore in the probe molecule is inhibited by HBQ1, resulting in no fluorescence emission

This study is based on the precise recognition and cleavage of mismatched nucleic acids by T7E Ⅰ. We employed nucleic acid mismatch enzyme detection (NMED) to detect CPV, with the reaction principle illustrated in Fig. 1. This study aims to develop and optimize a rapid, sensitive, specific, visual method for detecting CPV nucleic acids, providing a novel and practical technology for rapid onsite detection and control of parvovirus.

## Methods

### Reagents

E.Z.N.A.^TM^EndoFree Plasmid Midi Kit was purchased from Shanghai Solarbio Biotechnology Co., Ltd.The TwistAmpTM Basic Kit was purchased from TwistDx Bioscience Co., Ltd.The StarSpin Fast Virus DNA/RNA Kit and StarGreen safe nucleic acid dye were purchased from Beijing Genstar Biotechnology Co., Ltd. The 1×phosphate buffered saline, tryptone, yeast, sodium chloride, ampicillin sodium, and agarose were purchased fromWuhan Servicebio Co., Ltd.The HiScript III 1 st Strand cDNA Synthesis Kit, DNA Marker (DL500, DL2000), AceQ Universal U + Probe Master Mix V2, and RoomTemp Sample Lysis Kit were purchased from Nanjing Vazyme Biotechnology Co., Ltd. T7E I (10U/µL) was purchased from Beijing Viewolid Biotechnology Co., Ltd.The DNA Gel Extraction Kit was purchased from Beijing Tsingke Biotech Co., Ltd.

### Primer and probe synthesis

The VP2 gene sequence of the predominant CPV strain (GenBank: KF803636.1) was retrieved from GenBank. With DNASTAR software, this sequence was aligned with the VP2 gene sequences of CPV-2 A, CPV-2B, and CPV-2 C subtype strains. Conserved regions were identified and imported into Oligo7 software for the design of three pairs of RPA and qPCR primers, two sites and two lengths at each site of the fluorescent probe (including two base mutations). All primers and probes were synthesized by Shanghai Sangon Biotech Co., Ltd. (Table 1). After synthesis, the primers and probes were diluted to 10 µM and stored at −20 °C for subsequent use.

**Table 1:**
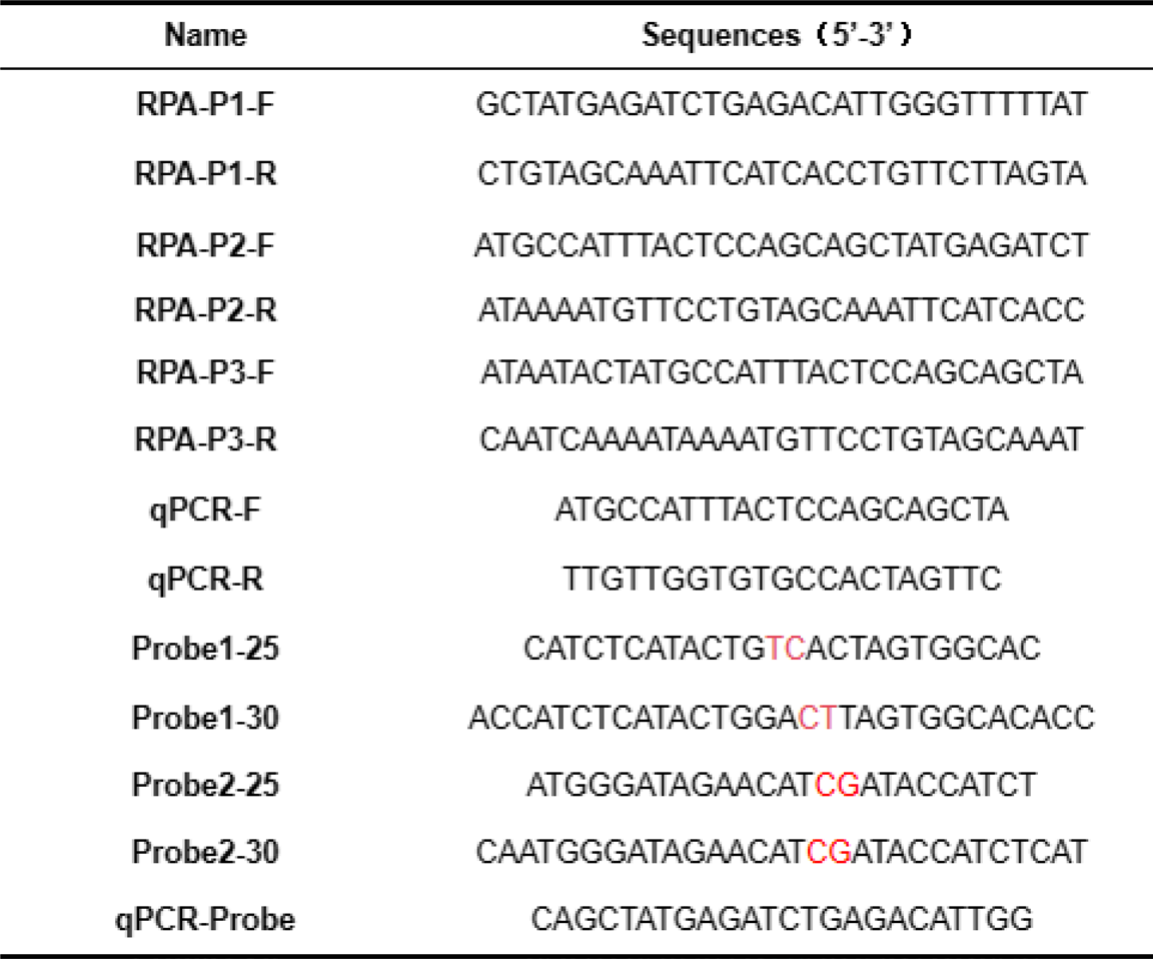
Primer and probe nucleotide sequences

### Standard virus acquisition and CPV clinical sample preparation

The standard viruses used in this study were canine distemper virus (CDV), canine parainfluenza virus (CPIV), CPV, canine adenovirus type 1 (CAV1), and canine coronavirus (CCV), which were provided by Zhongmu Biopharmaceutical Co., Ltd. According to the manufacturer’s instructions, the nucleic acids of the virus were extracted with the StarSpin Fast Virus DNA/RNA Kit, and then the RNA virus extracts were subjected to HiScript III 1 st Strand cDNA Synthesis Kit reverse transcription to cDNA.In addition, 32 suspected CPV samples were provided by Baole Pet Hospital and Ruipai Pet Hospital in Anyang city. DNA was extracted from the samplesvia the RoomTemp Sample Lysis Kit according to the manufacturer’s protocol. The extracted DNA was collected and stored at −20 °C for future use.

### pcDNA3.1-VP2 plasmid extraction and standard preparation

A total of 500 µL of *E. coli* culture containing the pcDNA3.1-VP2 plasmid was inoculated from the laboratory into 50 mL of LB liquid medium containing ampicillin. The mixture was incubated at 37 °C with shaking at 220 rpm for 10 h. Then, the culture was centrifuged at 3500 rpm for 10 min at room temperature, and the bacterial pellet was collected. The plasmids were extracted with the E.Z.N.A. ^TM^ EndoFree Plasmid Midi Kit according to the manufacturer’s instructions. After the concentration and purity were determined, the plasmid concentration was substituted into the formula to calculate the plasmid copy number [23].The extracted plasmid concentration was determined to be 518 ng/µL, with a plasmid size of 7176 bp. The copy number was calculated via a formula, and the plasmid was diluted to 10^7^ copies/µL as a positive standard.

### NMED detection platform

The NMED detection platform combines RPA amplification, nucleic acid hybridization and enzyme digestion.

47.5 µL RPA mixture was added to a 1.5 mL tube: 29.5 µL primer-free reconstitution buffer, 2.4 µL primer F (10 µM), 2.4 µL primer R (10 µM), 1 µL sample/plasmid (20 ng/µL), and 47.5 µL nuclease free water. The mixture was vortexed and spun briefly. The reaction mixture was added to a TwistAmp Basic reaction mixture, which was pipetted to mix. Add 2.5 µL of MgOAc (280 mM) and mix well to start the reaction. The mixture was incubated at 39 °C for 20 min. After 20 min, the RPA reaction products were identified on agarose gels.

2 µL the RPA reaction products were added and mixed with the appropriate proportion of 10×T7E I buffer and CPV probes. The samples were subsequently denatured, annealed, and renatured (98 °C for 3 min, 98 ~ 85 °C for 2 °C/s, and 85 ~ 25 °C for 0.2 °C/s). Then, 2 µL of T7E I was added, and the mixture was incubated at 37 °C for 15 min. The reaction was visualized via UV light. After the addition of T7E I, the mixture was directly placed into a fluorescence reader (Hangzhou Ruicheng Instrument Co., Ltd.), and the device automatically determined the result within 10 min.

### Polyacrylamide gel electrophoresis analysis

To prepare a 30% acrylamide gel with an acrylamide-to-bisacrylamide ratio of 29:1, follow these steps: Measure 1.5 mL of acrylamide stock solution into a suitable container. Add 1 mL of 5× TBE buffer, 85 µL of 10% ammonium persulfate (APS), and 3.8 µL of TEMED sequentially. Adjust the final volume to 4 mL using nuclease free water. Thoroughly mix the solution by gentle inversion to ensure homogeneity, taking care to avoid introducing excessive air bubbles. Immediately pour the mixture into the gel casting apparatus. Insert a clean sample comb without trapping air bubbles and allow the gel to polymerize undisturbed at room temperature for 30 min. Once polymerized, carefully remove the comb vertically to form well-defined sample wells. Mount the gel cassette into the electrophoresis tank, ensuring proper alignment. Fill the inner chamber with 1× TBE running buffer until the wells are completely submerged. Load the prepared samples into the wells using a micropipette. Fill the outer chamber of the electrophoresis tank with 1× TBE buffer to establish a continuous buffer system. Connect the electrophoresis tank to a power supply, ensuring the correct polarity (anode to cathode). Set the power supply to a constant voltage of 100 V and initiate electrophoresis for a duration of 40 min. When the target bands have migrated to approximately two-thirds of the gel length. After electrophoresis, carefully remove the gel from the cassette and transfer it to a staining tray containing a solution prepared with 10× SYBR Green I (diluted to the appropriate working concentration in buffer). Incubate the gel with gentle orbital shaking at room temperature for 1 h to allow dye binding. Following staining, transfer the gel to an imaging system (FIRE READER XS D-55-26.M, UVITEC Ltd., UK). Visualize the DNA bands under ultraviolet (UV) light illumination, where the stained bands will appear as distinct.

### Real-time PCR detection

TaqMan real-time qPCR detection of the CPV gene was carried out via a QuantStudio 6 Flex (Life Technologies) according to the AceQ Universal U + Probe Master Mix V2 recommended procedure described with the following modifications. Briefly, single-tube PCRs were prepared to contain 10 µL of 2×AceQ Universal U + Probe Master Mix V2, 0.5 µL of primer F, 0.5 µL of primer R, 0.2 µL of probe, 2 µL of DNA sample, and nuclease free water up to 20 µL. The amplification conditions used were an initial denaturation step of 95 °C for 5 min, followed by 45 cycles of 95 °C for 10 s and 60 °C for 30 s, with final cooling at 37 °C. Fluorescence information was collected at a 60 °C annealing extension per cycle.

### Determination of efficiency of the NMED method

The DNA/cDNAs of CDV, CPIV, CPV, CAV1 and CCV were extracted as templates, nuclease free water was used as a negative control to react according to the established NMED method, and the reaction products were detected by UV light and a fluorescence reader. Moreover, appropriate amounts of reaction products were taken for fluorescence quantitative analysis via a multi-Mode Microplate Reader (Synergy H1, American BioTek Instrument Co., Ltd.). The specificity of the established NMED method was verified.

A 10-fold serial dilution of the standard plasmid pcDNA3.1-VP2 was performed to obtain different concentrations of plasmid template at 10^7^ copies/µL, 10^6^ copies/µL, 10^5^ copies/µL, 10^4^ copies/µL, 10^3^ copies/µL, 10^2^ copies/µL, 10^1^ copies/µL and 10^0^ copies/µL. The CPV standard virus was used as a positive control, and nuclease free water was used as a negetive control. Reactions were carried out according to the established NMED method (Refer to the previous text “NMED detection platform”). After the reaction, the reaction products were subjected to UV light and detected with a fluorescence reader. Simultaneously, 5 µL of each reaction product was subjected to agarose gel electrophoresis to determine the limit of detection of copy number. 20 µL reaction products were then used for fluorescence quantification analysis via a microplate reader. The sensitivity of the NMED method was determined on the basis of the lowest copy number detected.

Three pcDNA3.1-VP2 standard plasmids with different concentrations (10^6^ copies/µL, 10^4^ copies/µL and 10^2^ copies/µL) were used as templates, and the detection was repeated 3 times for each concentration, with the CPV standard virus as a positive control and nuclease free water as a negative control. The repeatability and stability of the NMED method were verified.

### Visual inspection analysis

Visual detection was performed with UV light and a fluorescence reader. UV light is directly used for the End_point visualization fluorescence-based NMED detection of CPV. After the hybridization product of nucleic acid and the fluorescent probe is added to the T7E I, the single tube is immediately placed into the fluorescence reader at 37 °C, and rapid interpretation can be completed within 10 min, with positive results showing red light and negative results showing green light.

### Statistical analysis

ImageJ was used for grayscale analysis to analyze the T7E I digestive efficiency under different conditions. These data were analyzed according to previously described methods [24]. All the statistical analyses and graphing were performed via GraphPad Prism 6.01. The analyses were performed viaStudent’s t-test. The data are shown as the mean ± standard deviation (SD). All the results were generated from at least three independent biological replicates of each experiment.

## Results

### Development of the NMED assay for CPV detection

**Fig. 2:**
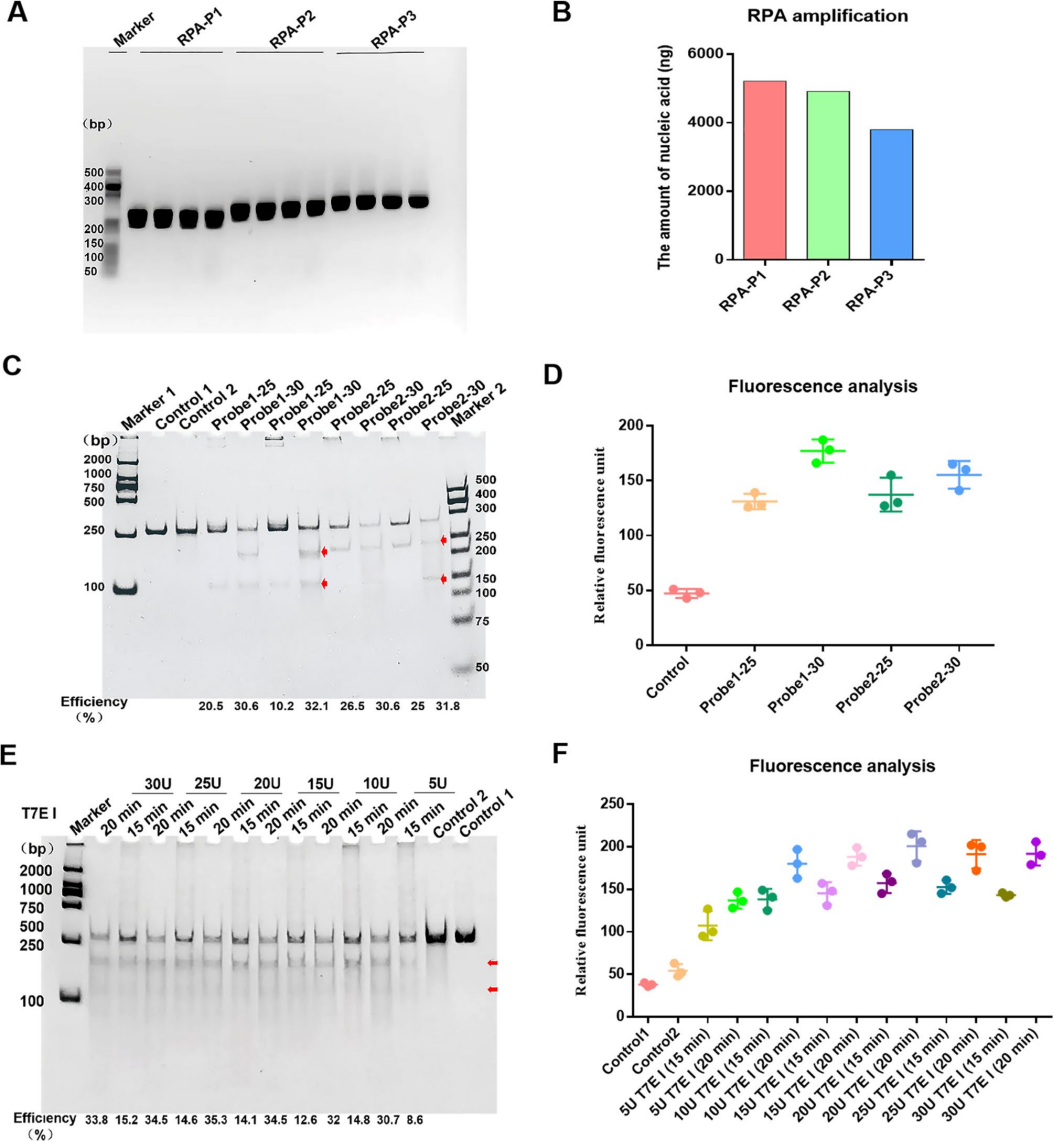
Establishing an NMED assay for detecting CPV. **A** The Amplification products of 3 primer pairs (RPA-P1, RPA-P2, and RPA-P3) were analyzed by nucleic acid electrophoresis. **B** The yield of RPA-amplified products was quantified. **C** Two different lengths (25 bp, 30 bp) of probe 1 and probe 2 were hybridized to the VP2 gene for T7E I recognition. The digestion efficiency was evaluated via PAGE and calculated, with cleaved bands indicated by red arrows. **D** The binding efficiency of the probes to the VP2 gene was determined via a Multi-Mode Microplate Reader. **E** Hybridized products were digested with varying T7E I concentrations (5 U, 10 U, 15 U, 20 U, 25 U, or 30 U) for 15–20 min. The red arrows indicate enzyme-cleaved bands. **F** The intensity of the fluorescence signal was measured via a Multi-Mode Microplate Reader following T7E I digestion

First, we screened optimal RPA primers for CPV. The gel electrophoresis results of the RPA products revealed that RPA-P1 yielded the most prominent band (Fig. 2A), and quantification via an ultramicro-ultraviolet spectrophotometer (NanoDrop™ One, Thermo Fisher) revealed that RPA-P1 yielded the highest amount of amplicon (Fig. 2B). Next, we evaluated fluorescent probes for efficient binding to the RPA products. The T7E I digestion assays demonstrated that all four tested probes could bind to the RPA products and be specifically recognized by T7E I. Among these, the 30-base probe (probe 1–30) exhibited the highest binding efficiency, with a digestion efficiency of 32.1% (Fig. 2C). The fluorescence measurements of the digested products using a multi-mode microplate reader further supported this. The 30-base probes were significantly superior to the 25-base ones, and probes 1–30 showed the highest fluorescence intensity, indicating the highest binding efficiency with the RPA products and the highest release of fluorescence (Fig. 2D).Subsequently, we optimized T7E I concentration and digestion time. The T7E I digestion results showed that the amount of digested product initially increased with T7E I cocentration up to 20 U, after which it plateaued. The 20 min digestion was more effective than 15 min (Fig. 2E). Corresponding fluorescence measurements mirrored these findings. The fluorescence initially reached a plateau faster with increasing enzyme concentration, peaking at 20 U with no significant increase thereafter, and the 20 min digestion was also superior to 15 min (Fig. 2F). Overall, the optimal conditions for the NMED method were determined as follows: the RPA primer RPA-P1, the hybridization probes 1–30, and the T7E I concentration 20 U, which collectively achieved the best detection performance.

### End-point visualization via the NMED assay

**Fig. 3:**
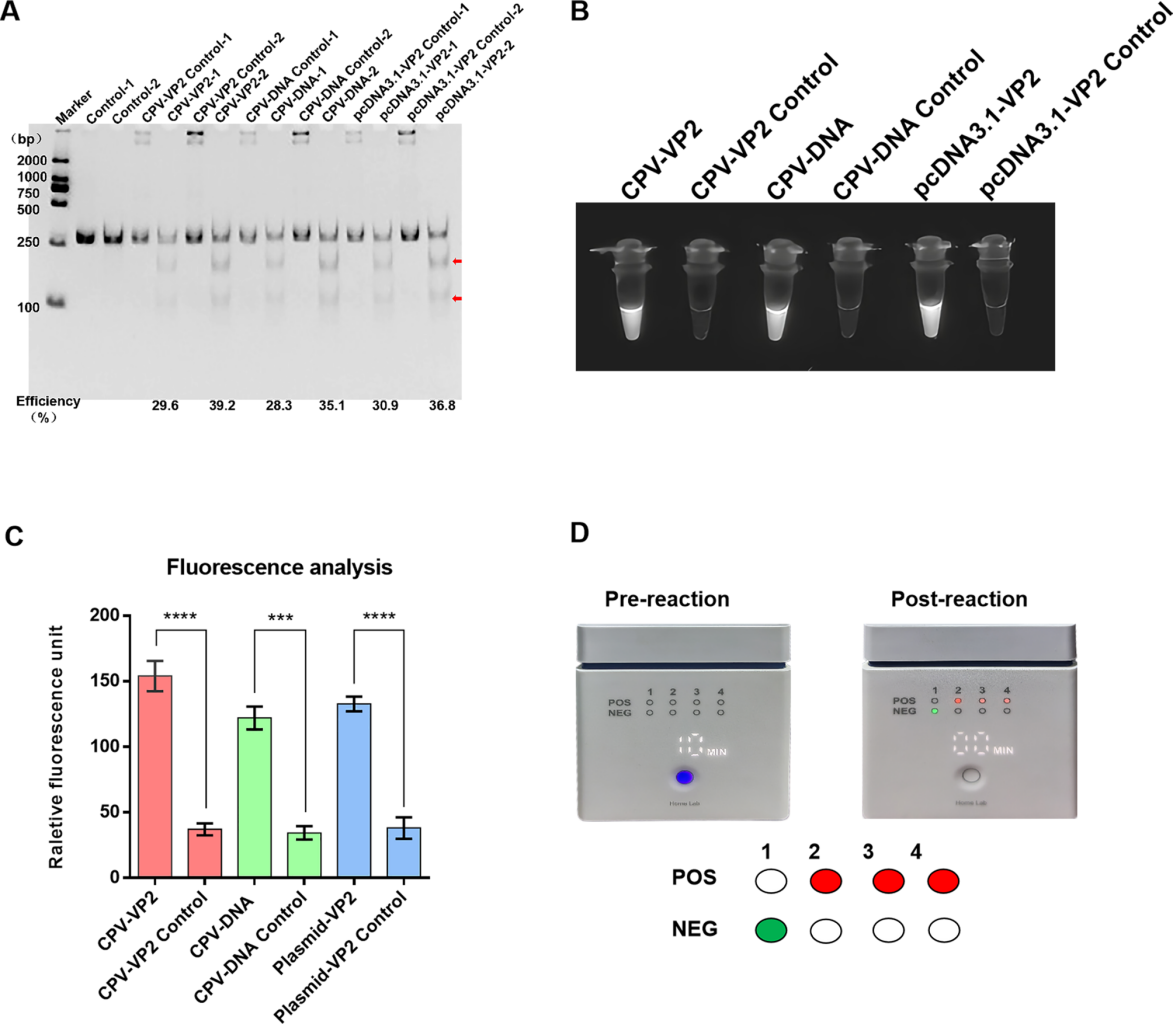
End-point visualization fluorescence-based NMED assay of the CPV gene.** A** Probes 1–30 were hybridized with three samples (CPV-VP2, CPV-DNA, and plasmid-VP2) for T7E I recognition and enzyme digestion. The enzyme digestion levels were determined via PAGE and quantified. The red arrows indicate bands cleaved by the enzyme. **B** The fluorescent signals of different samples were observed visually. **C** The fluorescence intensity was measured via a Multi-Mode Microplate Reader. ***:0.001 < *P* < 0.0001, ****:*P* < 0.0001. **D** The test combined a Fluorescence reader and fluorescence intensity assay. 1, 2, 3, 4 represent reaction wells in the Fluorescence reader device, and they were respectively the negative control, CPV-VP2, CPV-DNA, and plasmid-VP2. In the fluorescence reader results, POS (positive) is represented by red circles, whereas NEG (negative) is represented by green circles. According to the results of the fluorescence intensity assay, a bluer box color indicates higher abundance of CPV

To validate the feasibility of the NMED method for visualizing CPV detection, we assayed three target-containing samples, pcDNA3.1-VP2 standard plasmid, CPV viral DNA, and CPV VP2 gene under optimized reaction conditions. The T7E I digestion results showed efficient cleavage of products from all three samples, the enzyme digestion efficiency was above 28.3%. (Fig. 3A). Subsequent visualization via fluorescence reader and UV light revealed that, in contrast to the undigested control, all three samples with T7E I digestion products emitted distinct white fluorescence under UV light (Fig. 3B). Quantitative analysis confirmed significantly stronger fluorescence signals in digested samples compared to controls (*P* < 0.001), consistent with the UV light results (Fig. 3C). Furthermore, the real-time fluorescence monitoring during T7E I digestion enabled rapid result readout within 10 min (Fig. 3D). Collectively, these findings demonstrate the feasibility of NMED for end-point visualization of CPV detection.

### Specificity of the NMED assay

**Fig. 4:**
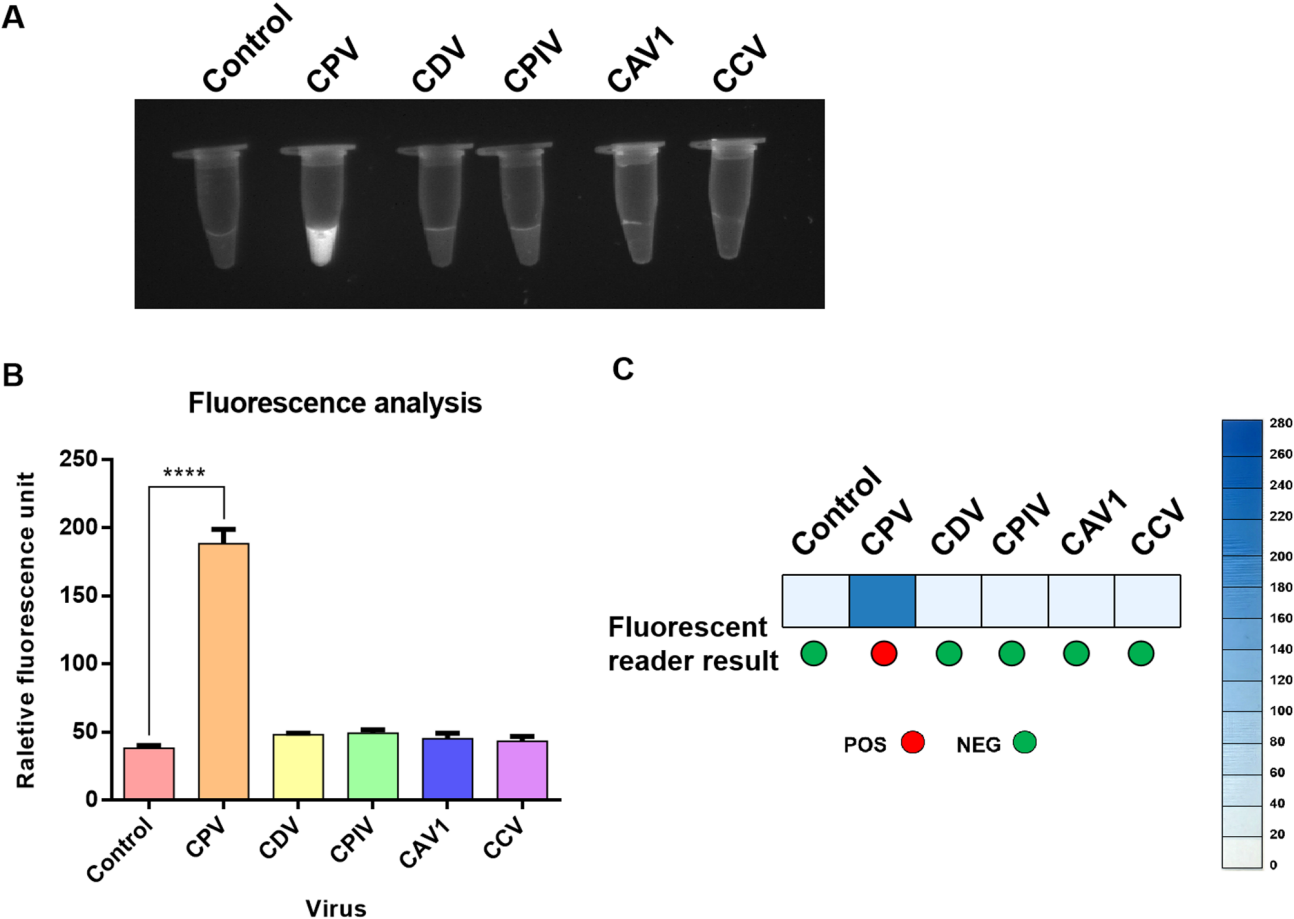
Determination of the specificity of the NMED assay for CPV. End_point visualization fluorescence-based NMED detection of CPV, CDV, CPIV, CAV1 and CCV. Control: no sample added. **A** The distinct fluorescent signals across the different viruses were observed visually using UV light. **B** Quantification using a Multi-Mode Microplate Reader showed significant differences in fluorescence signal intensity, ****: *P* < 0.0001. **C** The specificity was evaluated via both Fluorescence reader and fluorescence intensity assay, revealing positive responses. The results demonstrated that only the CPV samples yielded a positive reaction. In the fluorescence reader results, POS (positive) is represented by red circles, whereas NEG (negative) is represented by green circles. According to the results of the fluorescence intensity assay, a bluer box color indicates higher abundance of CPV

To evaluate the specificity of the NMED method for CPV detection, we applied the assay to CPV, CDV, CPIV, CAV1, and CCV separately. The results were assessed by both fluorescence reader and UV light. Under UV light, only CPV exhibited a white fluorescent signal, while the other viruses did not present a significant fluorescent signal (Fig. 4A). The fluorescent signal analysis more clearly revealed these results. CPV exhibited significantly higher intensity compared to the control (*P* < 0.0001). However, the intensities of the other viruses were essentially the same as those of the control, showing no difference (Fig. 4B). Consistent with this, fluorescence reader detection specifically indicated CPV (Fig. 4C). These results demonstrate the NMED assay is highly specific for CPV without cross-reactivity with other viruses.

### Sensitivity of the NMED assay

**Fig. 5:**
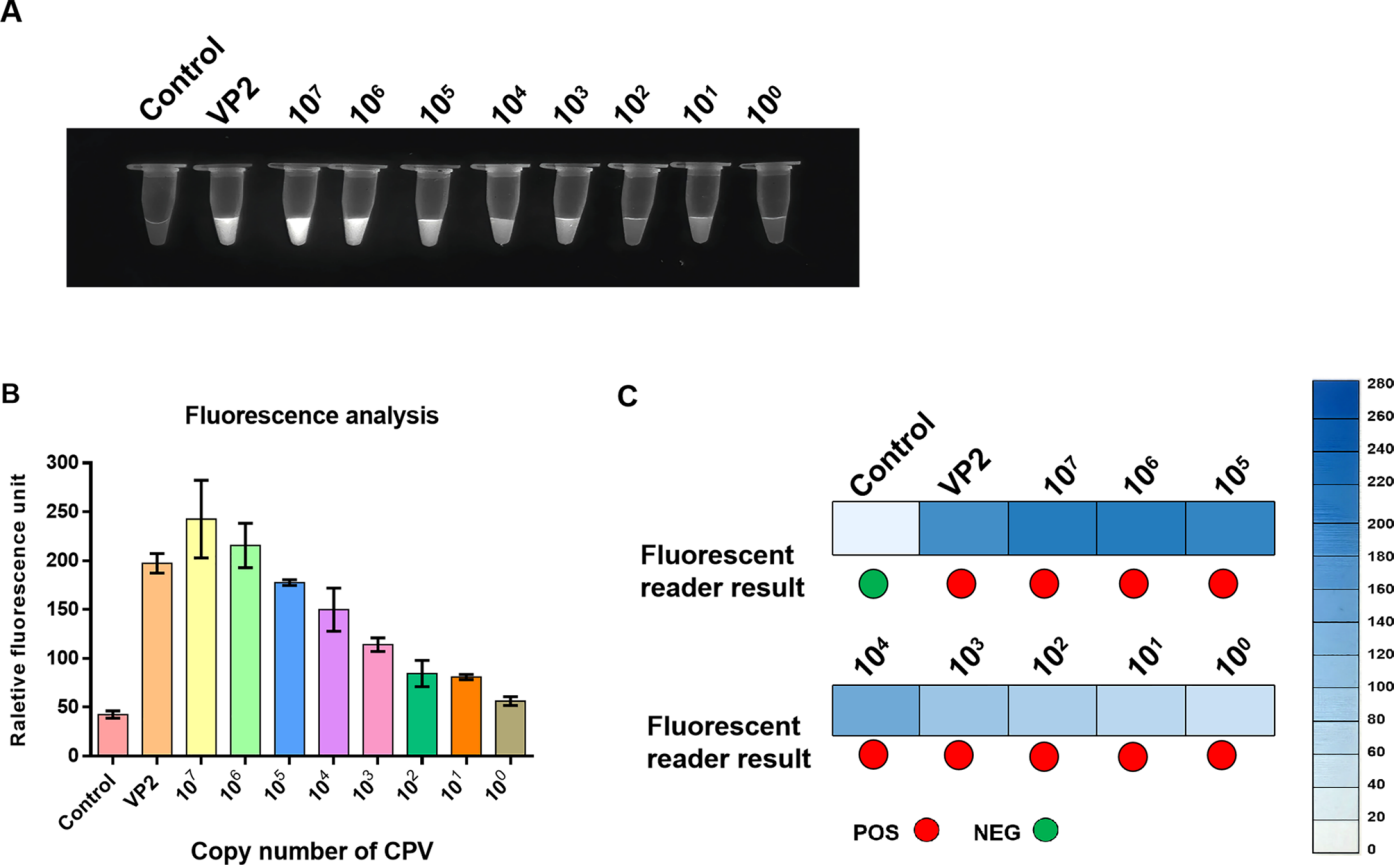
Determination of the sensitivity of the NMED assay. End_point visualization fluorescence-based NMED detection of VP2 plasmid dilutions. VP2 plasmids were tested at concentration ranging from 10^7^ to 10^0^ copies per reaction; control: no sample added; VP2: VP2 gene. **A** The fluorescent signal in different VP2 plasmid dilutions were observed visually using UV light. **B** Quantification using a Multi-Mode Microplate Reader showed differences in fluorescence signal intensity. **C** The sensitivity was evaluated via both Fluorescence reader and fluorescence intensity assay, revealing concentration-dependent positive responses. In the fluorescence reader results, POS (positive) is represented by red circles, whereas NEG (negative) is represented by green circles. According to the results of the fluorescence intensity assay, a bluer box color indicates higher abundance of CPV

To evaluate its sensitivity for CPV detection, we used the NMED to detect 10-fold serial dilutions of the pcDNA3.1-VP2 standard plasmid (10^7^~ 10^0^ copies per reaction). The results were assessed via a fluorescence reader and UV light. UV light detection revealed that the fluorescence intensity gradually decreased with lower DNA copy numbers, with a detection limit of 10 copies or more (Fig. 5A). For comparison, a Multi-Mode Microplate Reader measured fluorescence intensity of the reaction products, which was significantly higher than the control when the DNA copy numbers were 10 or more (Fig. 5B). Additionally, the sensitivity evaluation using a fluorescence reader showed accurate detection of 1 or more (Fig. 5C). The results indicate the NMED-based CPV system has a detection range of 1–10 copies or more, with the fluorescence reader being faster and more sensitive than UV light.

### Repeatability of the NMED assay

**Fig. 6:**
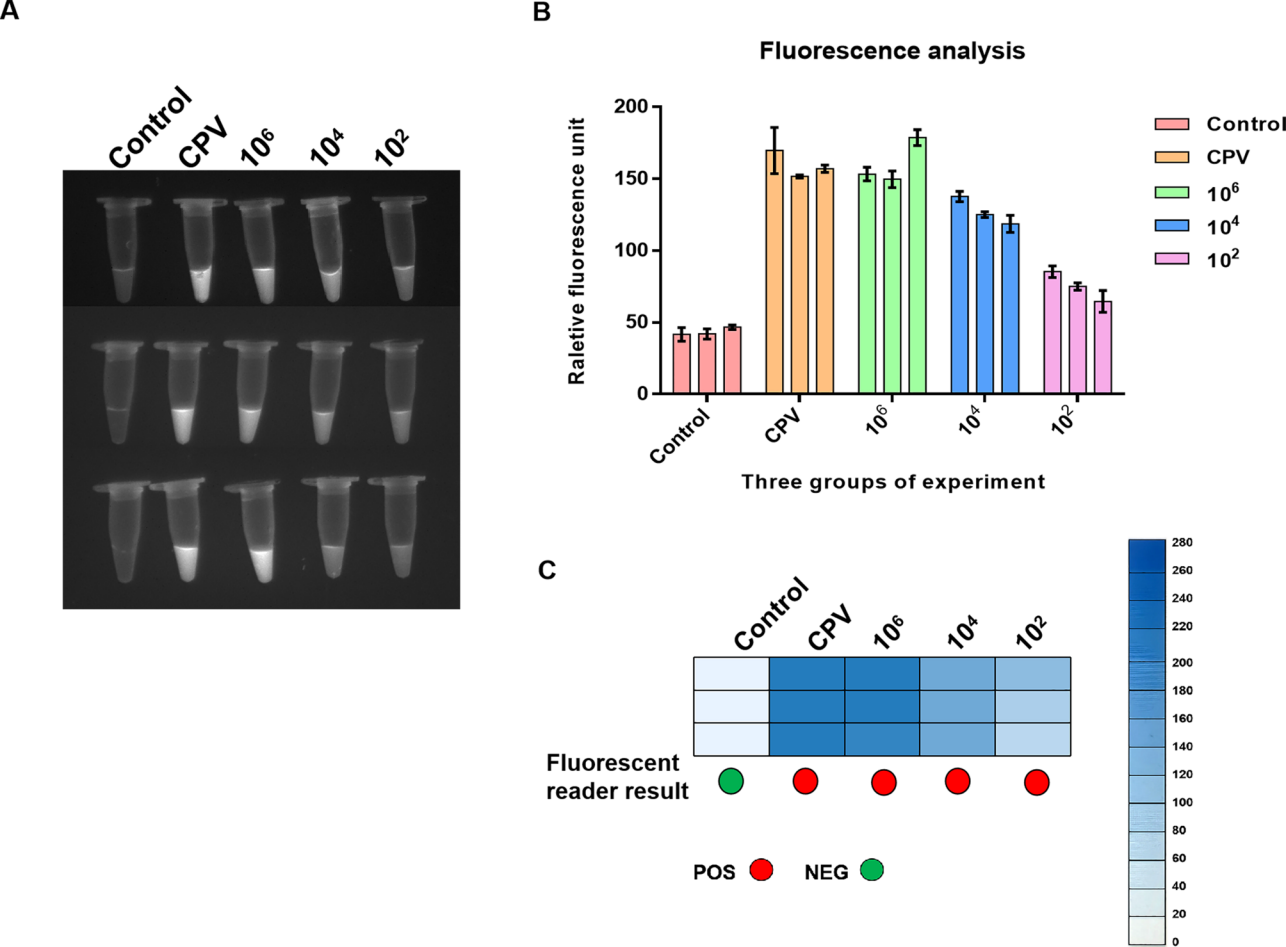
Determination of the repeatability of the NMED assay. End_point visualization fluorescence-based NMED detection of VP2 plasmid repeatability. Three concentrations of VP2 plasmids (10^6^, 10^4^, and 10^2^ copies per reaction) were tested in triplicate; control: no sample added; CPV: CPV standard virus. **A** The fluorescent signals in different VP2 plasmid dilutions were observed visually using UV light. **B** Quantification using a Multi-Mode Microplate Reader showed differences in fluorescence signal intensity. **C** the repeatability was evaluated via both Fluorescence reader and fluorescence intensity assay, revealing coincident positive reactions in triplicates for all VP2 dilutions. In the fluorescence reader results, POS (positive) is represented by red circles, whereas NEG (negative) is represented by green circles. According to the results of the fluorescence intensity assay, a bluer box color indicates higher abundance of CPV

To evaluate CPV detection repeatability, triplicate assays were performed using pcDNA3.1-VP2 standard plasmid at three concentrations (10^6^, 10^4^, and 10^2^ copies/µL). End-point visualization showed clearly detectable and highly consistent results in three replicates (Fig. 6A). The fluorescence signal intensities analyzed further confirmed consistency, with no significant differences between samples of identical DNA copy numbers (Fig. 6B). The repeatability assessed via a fluorescence intensities yielded concordant results, and showing comparable signal intensities for matched DNA concentrations (Fig. 6C). Collectively, these results demonstrate the stable repeatability of the NMED method.

### Validation of the NMED assay for clinical isolates

**Fig. 7:**
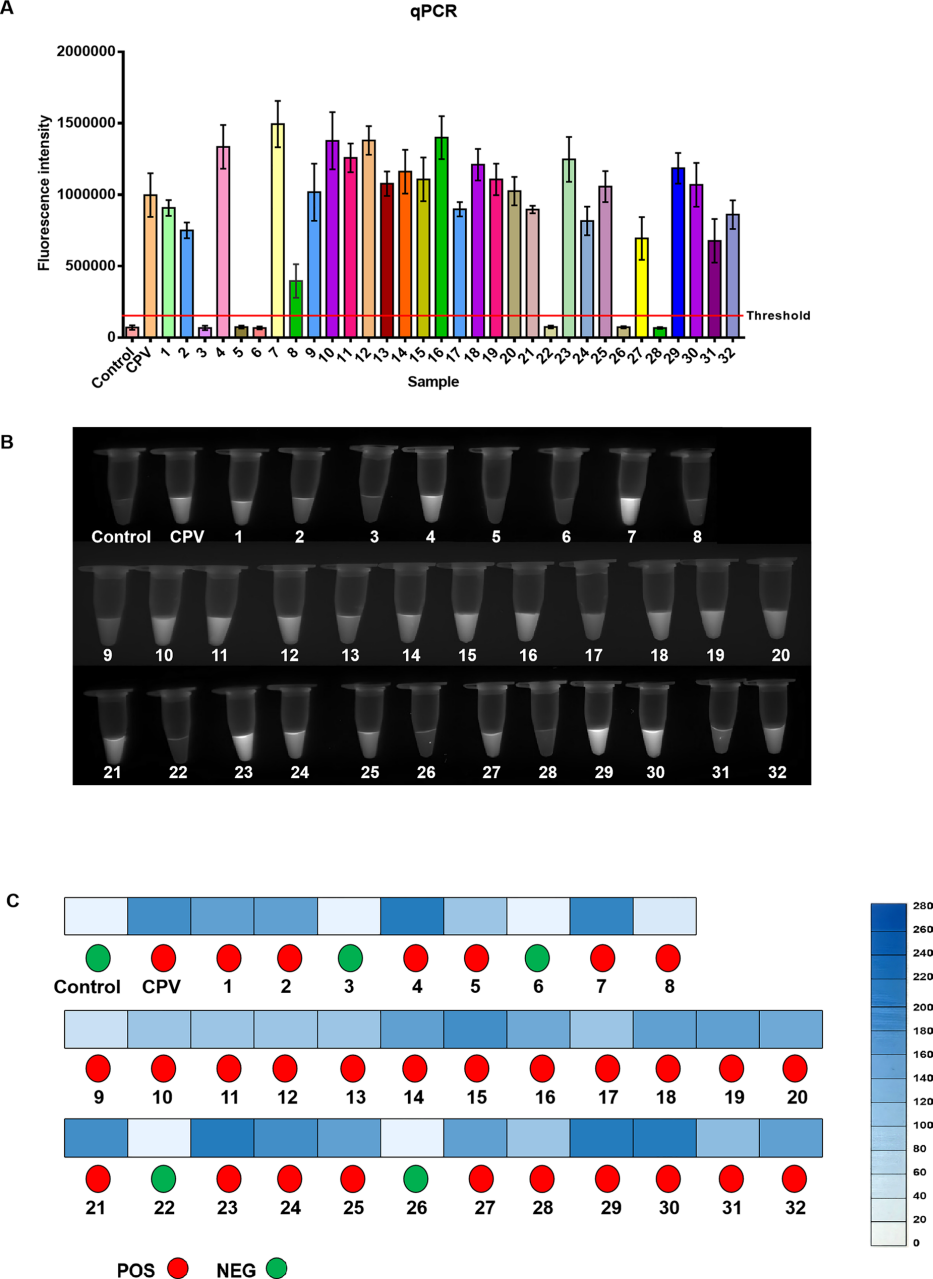
Determination of the clinical isolatesvia the NMED assay. **A** Diagnostic results of 32 CPV clinical isolates by qPCR. **B** The fluorescent signals of 32 clinical isolates were observed visually using UV light. Control: no sample added; CPV: CPV standard virus. **C** 32 clinical isolates were evaluated via both Fluorescence reader and fluorescence intensity assay. In the fluorescence reader results, POS (positive) is represented by red circles, whereas NEG (negative) is represented by green circles. According to the results of the fluorescence intensity assay, a bluer box color indicates higher abundance of CPV. Control: no sample added; CPV: CPV standard virus

We furthe evaluatedr the NMED’s performance in diagnosing CPV in clinical isolates, using qPCR as the gold standard. Among 32 clinical samples, the qPCR results identified 26 positive and 6 negative samples(Fig. 7A). NMED analysis was performed using both a fluorescence reader and UV light. UV light-based fluorescence detection yielded 23 positive and 9 negative samples (Fig. 7B), with an accuracy rate of 90.63%. Additionally, for the fluorescence reader, samples were incubated at 37 °C for 10 min after of T7E I addition. The method detected 28 positive and 4 negative samples (Fig. 7C), with an accuracy rate of 93.75%, demonstrating high sensitivity but a tendency for false-positives. These results confirm the utility of NMED for CPV in clinical isolates (Table 2).

**Table 2:**
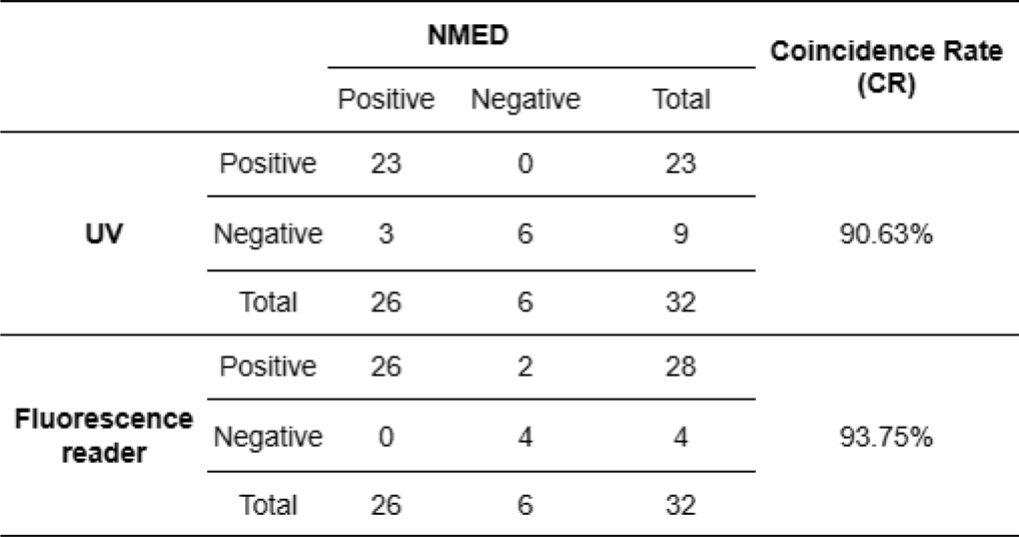
Clinical validation of the NMED assay for CPV

## Discussion

Parvoviruses pose a serious threat not only to the health of livestock, causing significant economic losses but also to human health. Therefore, rapid, specific, and sensitive virus detection techniques are crucial for timely identification of parvovirus, preventing its spread, avoiding cross-infection with humans, and providing accurate epidemiological information during outbreaks. Traditional parvovirus detection methods, such as cultivation, morphological observation, and biochemical identification, have drawbacks, including time-consuming procedures, poor specificity, and low sensitivity [25]. RT‒qPCR, considered the “gold standard” for nucleic acid detection, requires expensive instruments and equipment, has a long detection time, and may experience cross-contamination [26]. Colloidal gold technology, despite its convenience and speed, has lower sensitivity, lower specificity, and inability to quantify than nucleic acid detection [27]. Detection methods based on isothermal amplification technology can efficiently amplify the target gene at a single temperature without relying on precise temperature control equipment. This method has the potential to become POCT. Among them, isothermal amplification techniques based on LAMP and RPA are mainstream and widely used for the detection of various viruses. These methods are characterized by their speed, high sensitivity, and wide detection range. Geng et al. [28] established a real-time RPA method for CPV-2. This method can successfully detect as few as 10 copies of CPV-2 at 38 °C. It does not cross-react with other viruses. The clinical sensitivity of real-time RPA detection is 100%, which is consistent with real-time PCR results. However, high false-positive rates and the complexity of reagents pose challenging technical issues. With the rapid development of nucleic acid detection technologies, isothermal nucleic acid amplification techniques, coupled with colorimetric methods, fluorescence methods, microfluidic chip technology, multiplex sequencing technology, and CRISPR/Cas technology, have been widely used in the field of life sciences, especially in viral detection, where they have developed rapidly [29]. Wang et al. [30] developed a novel FPV rapid detection method, LFD-RPA, which consists of specific primers, probes, and nucleic acid strips. This method can detect different subtypes of FPV within 15 min at 38 °C. Each reaction can detect more than 10^2^ copies of FPV, achieving a sensitivity one order of magnitude greater than that of PCR. Additionally, a series of CRISPR-Dx technologies are rapidly evolving. By utilizing the collateral cleavage activity of certain *Cas* proteins in conjunction with isothermal amplification techniques, these technologies not only exhibit rapid, efficient, and sensitive characteristics but also fulfill the POCT requirements by employing lateral flow dipsticks, reaction solution color changes, electrochemical biosensors, and other methods for laboratory-free applications. Wang T et al. [31] established a real-time or endpoint fluorescence detection method based on RPA-Cas12 to identify FPV. The fluorescence detection method took approximately 25 min in total, with a detection limit of 1 copy/µL and no cross-reactivity with other pathogens. The results of 60 clinical samples were consistent with the results of qPCR. Wei et al. [32] constructed an ERA-CRISPR/Cas12a system for the detection of PPV. This method can visually detect more than 3.75 × 10^2^ copies/µL of PPV under isothermal conditions at 37 °C and does not cross-react with other porcine viruses.While CRISPR-Dx technology has advanced rapidly, challenges remain in its clinical application. CRISPR-Dx faces limitations in crRNA design because of its dependence on PAM sites, and it is susceptible to off-target effects. Moreover, the utilization of the Cas protein to cleave ssDNA results in insufficient specificity and stability. Therefore, the development of novel detection systems or the refinement of existing technologies is crucial to ensure reliability and clinical applicability in viral detection.

In this study, we propose a novel method, termed NMED, based on characteristic fluorescent probes and T7E I recognition. In this NMED method, the required selection site of primers is large, the design is simple, and the application is easy. Our proposed method utilizes the T7E I endonuclease’s characteristics to identify the mutation site. It significantly enhances detection specificity by identifying the mutant base in the hybridized sequence of the specific targeted sample DNA and specific fluorescent antibody. Compared with CRISPR-Dx, the NMED method requires only basic temperature control equipment for the entire detection process, with a simpler probe design, superior specificity, greater efficiency, and greater ease of application. This method specifically targets CPV without cross-reactivity with other viruses, demonstrating strong specificity. The detection sensitivity approaches 1–10 copies or more, with good repeatability. Compared with that of qPCR, the detection concordance rate reached 90.63–93.75%. Our research further revealed that fluorescence readers can enhance the sensitivity of the NMED assay by 10-fold, offering greater convenience with direct result interpretation, but there is also a risk of false positives.

## Conclusions

The NMED method has been successfully applied in laboratories and veterinary clinics for the detection of CPV, and some results have been achieved. This method is easy to learn and use, can be quickly mastered, and saves time and testing costs. The NMED method has commercial potential and provides new ideas and technical approaches for the detection of parvovirus and other clinical pathogens.

## Supplementary Information

Supplementary Material 1.

Supplementary Material 2.

Supplementary Material 3.

Supplementary Material 4.

## Data Availability

The datasets used and/or analyzed during the current study are available from the corresponding author upon reasonable request.
