# Supplementary figures and images for: Establishment and application of a visual nucleic acid detection method for parvovirus
Source: BMC Vet Res. 2025 Oct 2;21:563. doi: 10.1186/s12917-025-04993-5 (PMC12492551; doi:10.1186/s12917-025-04993-5)

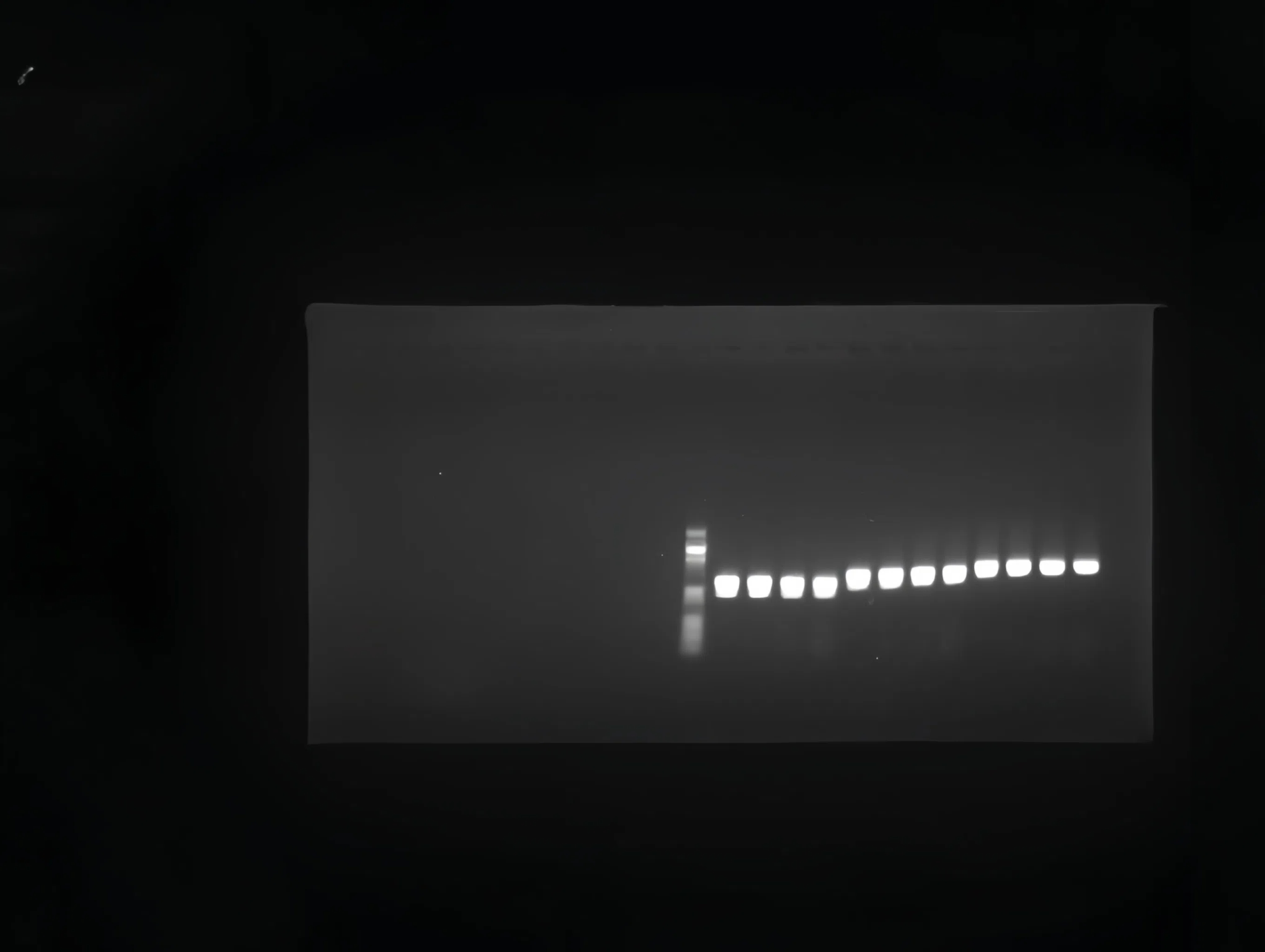

Supplement: Supplementary file 1 — Supplementary Material 1. [file 12917_2025_4993_MOESM1_ESM.tif]

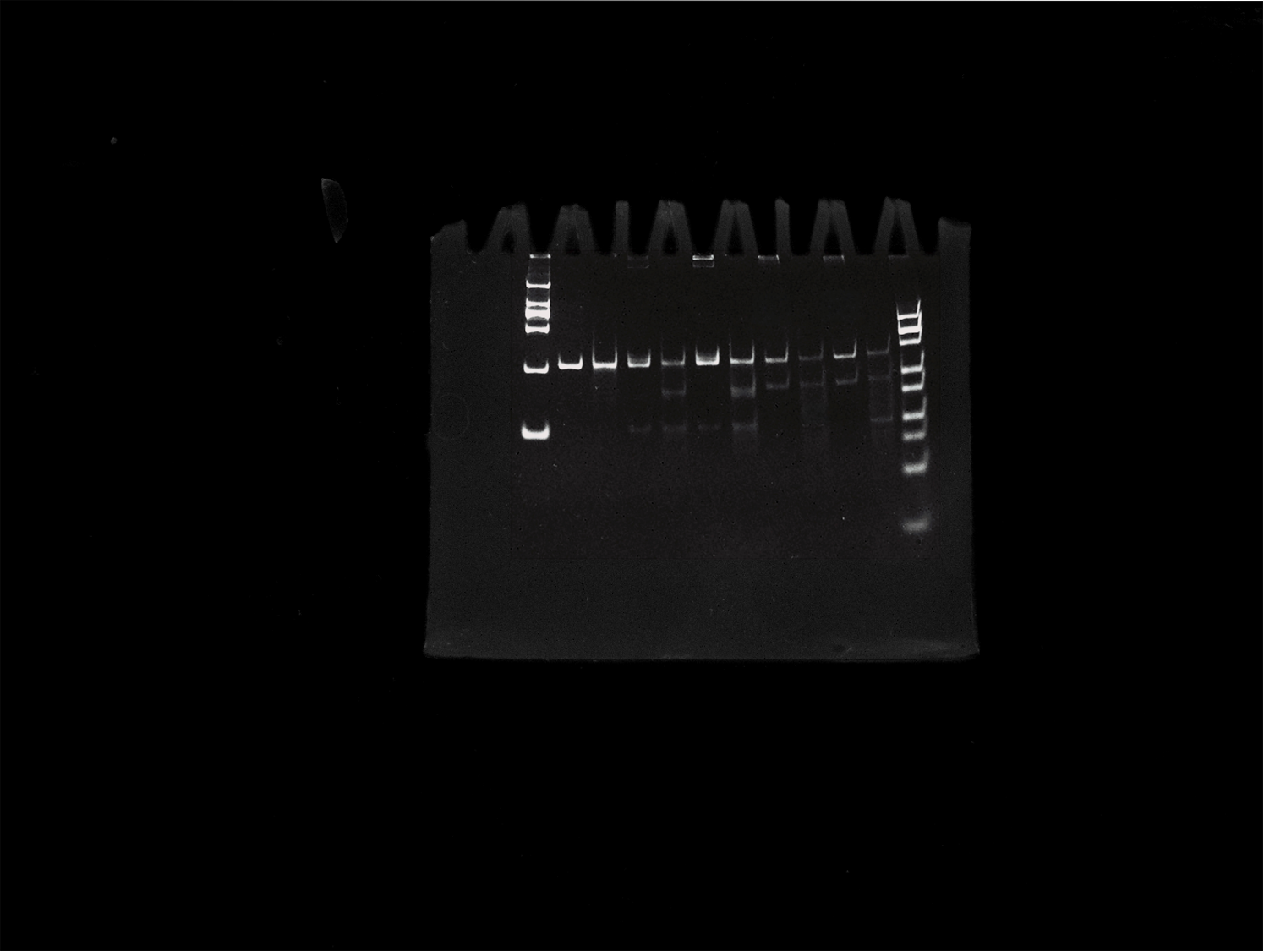

Supplement: Supplementary file 2 — Supplementary Material 2. [file 12917_2025_4993_MOESM2_ESM.tif]

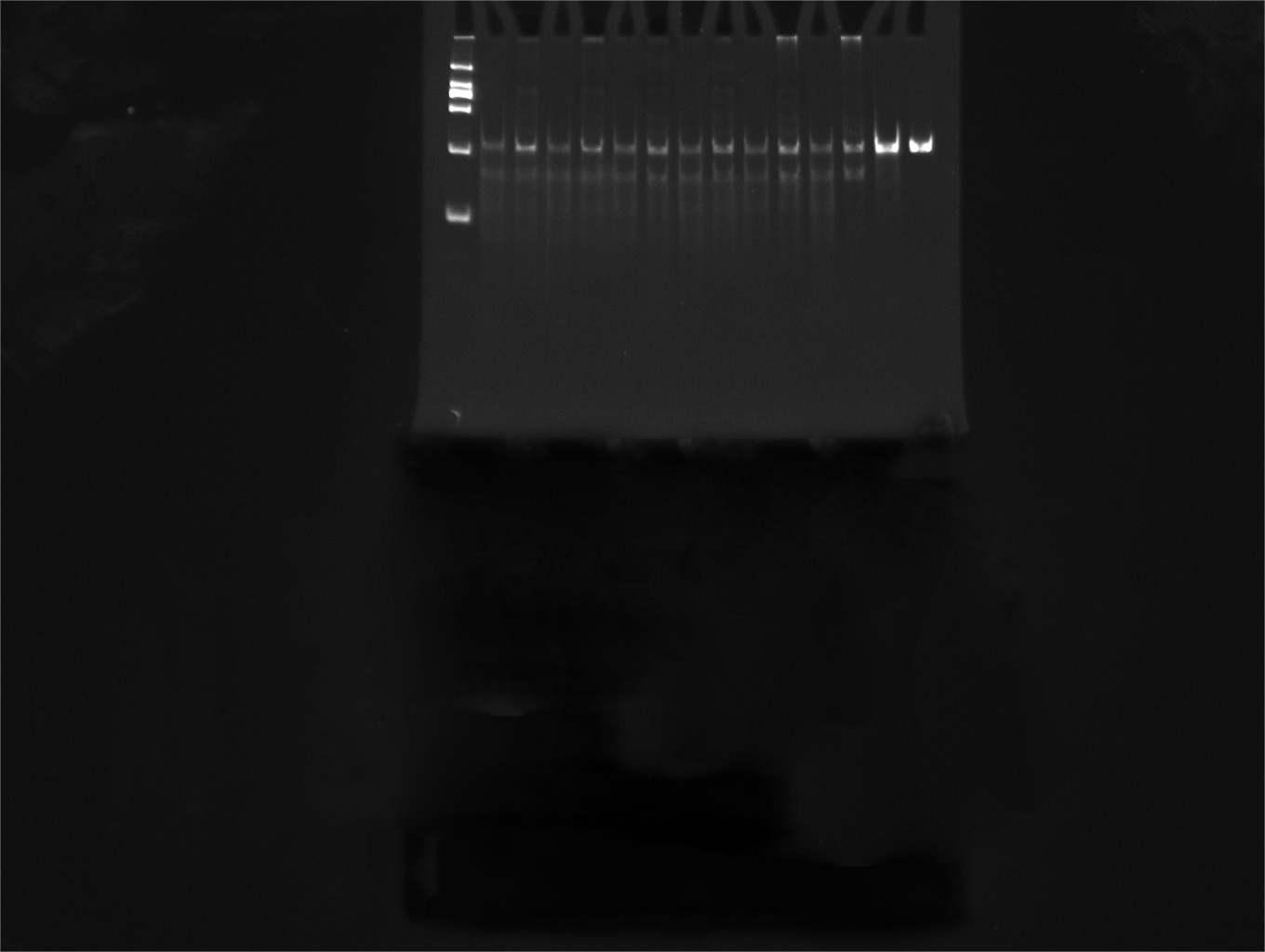

Supplement: Supplementary file 3 — Supplementary Material 3. [file 12917_2025_4993_MOESM3_ESM.tif]

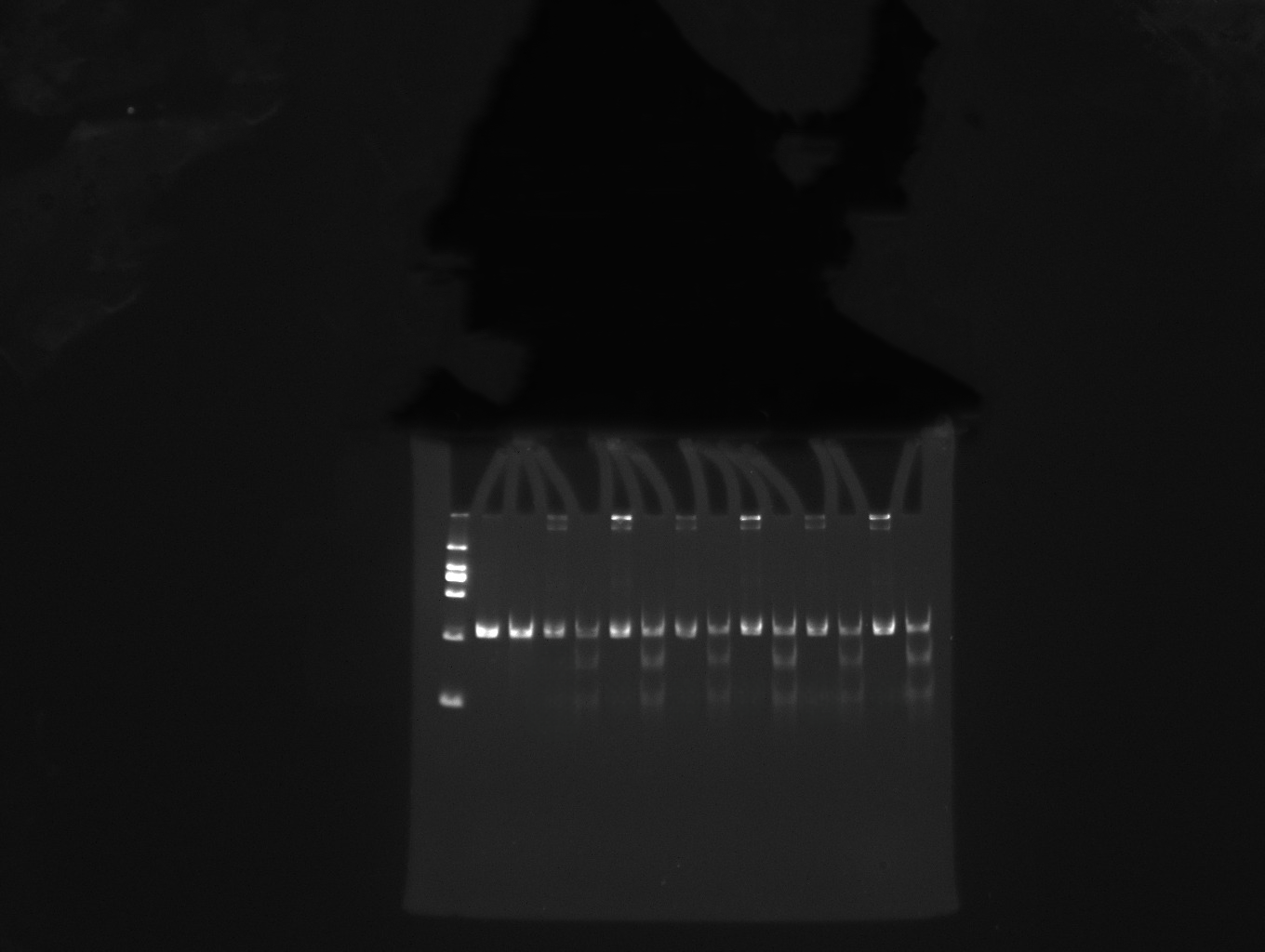

Supplement: Supplementary file 4 — Supplementary Material 4. [file 12917_2025_4993_MOESM4_ESM.tif]
